# Knowledge, attitude and behavior of mothers about prevention of childhood accidents and injuries in under-6-year-old children 

**Published:** 2019-08

**Authors:** Raheleh Soltani, Safiye Jahanmehr

**Affiliations:** ^*a*^Department of Health Education and Promotion, School of Health, Arak University of Medical Sciences, Arak, Iran.; ^*b*^Unit of Bio-Statistics, Health Chancellor, Tabriz University of Medical Sciences, Tabriz, Iran.

## Abstract

**Background:**

Knowledge, Attitude and Behavior of Mothers about Prevention of Childhood Accidents and Injuries in Under-6-year-old Children Background: injury and violence prevention in “healthy people project 2020”, has been considered as one of the most important factor to improve health promotion. Parents are directly responsible for the children health and can play an important role in preventing of childhood accidents and injuries. Purposes: The aim of this study was to assess knowledge, attitude and behavior of mothers in the prevention of childhood accidents.

**Methods:**

This cross-sectional study that was carried out on 450 mothers of children less than 6 years old referred to health centers in Tabriz, 2016. Data was collected using a validated questionnaire including demographic characteristic (6 items), knowledge (15 items), attitude (5items) and behavior (13items) of mothers about prevention accidents and injuries in children. SPSS Ver-16 statistical software was used to analyze the data. The significance level of α = 0/05 was considered for all tests.

**Results:**

The mean age of mothers was 28.2 (SD=5) and children 4.6 (SD=1.5). According to the results 24% of mothers were in higher education and majority of the mothers (88%) were housewives. About 26.5 of mothers had poor, 44.7% moderate and 28.8% good knowledge about preventing accidents and injuries in children. The mean (SD) attitude score was 8.4 (3.8) and 75.8% of mothers had high attitude. Mothers’ behavior related to childhood accidents were Poor (15%), average (35.8), and good (27.7%). The overall prevalence of injury among children was 38%. The most reasons for injury were falling (55.1%), burns (19.8%) and poisoning (8.4%). The knowledge (p<0.01) and attitude (p<0.05) had a meaningful relationship with the behavior of mothers about prevention of childhood accidents and injuries. There was statistically association between mothers knowledge and education level (P <0.001, R= 0.516).

**Conclusions:**

According to the results of this study, mothers’ knowledge and behavior was not desirable about prevention of childhood accidents and injuries. Health promotion interventions based on mothers training needs seem necessary. So it is important to increase families’ knowledge through community –based intervention.

**Keywords:**

Behavior, Knowledge, Accidents, Injuries, Children

